# Liraglutide ameliorates inflammation and fibrosis by downregulating the TLR4/MyD88/NF-κB pathway in diabetic kidney disease

**DOI:** 10.1152/ajpregu.00083.2024

**Published:** 2024-08-12

**Authors:** Linjing Huang, Tingting Lin, Meizhen Shi, Peiwen Wu

**Affiliations:** ^1^Department of Endocrinology, the First Affiliated Hospital, Fujian Medical University, Fuzhou, People’s Republic of China; ^2^Department of Endocrinology, National Regional Medical Center, Binhai Campus of the First Affiliated Hospital, Fujian Medical University, Fuzhou, People’s Republic of China; ^3^Clinical Research Center for Metabolic Diseases of Fujian Province, the First Affiliated Hospital, Fujian Medical University, Fuzhou, People’s Republic of China; ^4^Fujian Key Laboratory of Glycolipid and Bone Mineral Metabolism, the First Affiliated Hospital, Fujian Medical University, Fuzhou, People’s Republic of China; ^5^Diabetes Research Institute of Fujian Province, the First Affiliated Hospital, Fujian Medical University, Fuzhou, People’s Republic of China; ^6^Department of Endocrinology, Nanping First Hospital Affiliated to Fujian Medical University, Nanping, People’s Republic of China

**Keywords:** diabetic kidney disease, extracellular matrix, inflammation, liraglutide, TLR4/MyD88/NF-κB

## Abstract

Inflammation and fibrosis play important roles in diabetic kidney disease (DKD). Previous studies have shown that glucagon-like peptide-1 receptor (GLP-1R) agonists had renal protective effects. However, the mechanisms are not clear. The present study explored the effect of liraglutide (LR), a GLP-1R agonist, on the downregulation of glomerular inflammation and fibrosis in DKD by regulating the Toll-like receptor (TLR)4/myeloid differentiation marker 88 (MyD88)/nuclear factor κB (NF-κB) signaling pathway in mesangial cells (MCs). In vitro, rat MCs were cultured in high glucose (HG). We found that liraglutide treatment significantly reduced the HG-mediated activation of the TLR4/MYD88/NF-κB signaling pathway, extracellular matrix (ECM)-related proteins, and inflammatory factors. A combination of TLR4 inhibitor (TAK242) and liraglutide did not synergistically inhibit inflammatory factors and ECM proteins. Furthermore, in the presence of TLR4 siRNA, liraglutide significantly blunted HG-induced expression of fibronectin protein and inflammatory factors. Importantly, TLR4 selective agonist LPS or TLR4 overexpression eliminated the improvement effects of liraglutide on the HG-induced response. In vivo, administration of liraglutide for 8 wk significantly improved the glomerular damage in streptozotocin-induced diabetic mice and reduced the expression of TLR4/MYD88/NF-κB signaling proteins, ECM protein, and inflammatory factors in renal cortex. TLR4^−/−^ diabetic mice showed significant amelioration in urine protein excretion rate, glomerular pathological damage, inflammation, and fibrosis. Liraglutide attenuated glomerular hypertrophy, renal fibrosis, and inflammatory response in TLR4^−/−^ diabetic mice. Taken together, our findings suggest that TLR4/MYD88/NF-κB signaling is involved in the regulation of inflammatory response and ECM protein proliferation in DKD. Liraglutide alleviates inflammation and fibrosis by downregulating the TLR4/MYD88/NF-κB signaling pathway in MCs.

**NEW & NOTEWORTHY** Liraglutide, a glucagon-like peptide-1 receptor agonist (GLP-1RA), has renoprotective effect in diabetic kidney disease (DKD). In DKD, TLR4/MYD88/NF-κB signaling is involved in the regulation of inflammatory responses and extracellular matrix (ECM) protein proliferation. Liraglutide attenuates renal inflammation and overexpression of ECM proteins by inhibiting TLR4/MYD88/NF-κB signaling pathway. Therefore, we have identified a new mechanism that contributes to the renal protection of GLP-1RA, thus helping to design innovative treatment strategies for diabetic patients with various complications.

## INTRODUCTION

Diabetic kidney disease (DKD) is a microvascular complication of diabetes (1). At present, the global prevalence of DKD has risen sharply, seriously endangering human health. However, the pathogenesis of DKD is still undefined. The early pathological changes of DKD are glomerular hypertrophy, glomerular mesangial cell (MCs) proliferation, and extracellular matrix (ECM) progressive accumulation, which eventually leads to fibrosis in the later stage of DKD (2). The excessive production of ECM by MCs and the accumulation in the glomerular basement membrane and mesangial matrix are important factors that aggravate DKD damage. Another pathogenesis of DKD is inflammation (3). High glucose (HG) environment increases the secretion of inflammatory factors. The interaction between different inflammatory factors causes an inflammatory cascade reaction, leading to the thickening of the glomerular basement membrane, increases urinary protein excretion, and finally leads to end-stage renal disease (4). Therefore, reducing the oversecretion of ECM and inflammatory response in MCs is one of the early options for the treatment of DKD.

Glucagon-like peptide 1 (GLP-1), an incretin, stimulates glucose-dependent insulin secretion and promotes glucose metabolism through various physiological effects. GLP-1 receptor agonist (GLP-1RA), as a hypoglycemic agent, has drawn attention due to its additional improvement effect on the kidney (5–7). It has been reported that GLP-1RA improved the inflammatory response and oxidative stress in MCs (8). Bai et al. (9) reported that GLP-1RA liraglutide (LR) improved renal fibrosis in angiotensin II-induced rats by attenuating the protein levels of nicotinamide adenine dinucleotide phosphate oxidase 4 and interleukin-6 (IL-6) and downregulating the synthesis of fibronectin (FN), collagen I, and α-smooth muscle actin (α-SMA). We recently found that liraglutide reduced HG-induced ECM overproduction by increasing GLP-1 receptor expression in MCs (10). Therefore, GLP-1RA may bring renal benefits through different pathways.

Toll-like receptor (TLR), a transmembrane protein, delivers extracellular antigen recognition information intracellular and triggers an inflammatory response, and mediates immune response (11). In the kidney, the TLR4 is an important signaling molecule that mediates kidney inflammation and fibrosis (12). Increased TLR4 expression in DKD upregulates inflammatory response and releases the fibrosis-related factors in type 2 diabetes (T2DM) and its chronic complications (12–15). Therefore, TLR4 antagonists are targeted in the treatment of DKD. Previous studies reported that TLR4 signaling is involved in the protective effects of GLP-1RA in extrarenal organs/tissues (16–18). For instance, in the liver, both liraglutide and exenatide improved liver lesions by regulating the TLR4/nuclear factor κB (NF-κB) inflammatory pathway and oxidative stress in T2DM/nonalcoholic fatty liver disease (NAFLD) rats (16, 17). In the pancreas, exendin-4 inhibited lipotoxicity-induced oxidative stress by reducing the activation of TLR4 and NF-κB p65 subunit in β cells (18). However, the relationship between GLP-1RA and the TLR4 signaling pathway in DKD remains unclear. This study aimed to identify the possible role of liraglutide in improving inflammation and alleviating fibrosis in DKD mediated by TLR4 signaling pathways.

## MATERIALS AND METHODS

### Cell Cultures

Rat glomerular mesangial cell line (RMCs, Catalog No. YBC283) was purchased from Yubo Biotechnology Co., Ltd. (Shanghai, PR China) and cultured in DMEM (Gibco, Carlsbad, CA) containing 10% fetal bovine serum (FBS), 100 U/mL penicillin, and 100 mg/mL streptomycin in a 5% CO_2_ and 37°C incubator. The medium was replaced every 2 days. Cells at ∼80% confluence were growth-arrested in serum-free DMEM overnight before all treatments.

### d-Glucose and Liraglutide Treatments In Vitro and In Vivo

#### In vitro study.

Cells were treated with normal glucose (NG, 5.5 mM d-glucose), or 5.5 mM d-glucose and 19.5 mM mannitol (NG + M, the osmotic control), or HG (25 mM d-glucose) in the presence or absence of liraglutide (LR, 100 nM, Catalog No. HY-P0014, MedChemExpress) for 48 h. In some experiments, cells were treated with TAK242 (1 µM, Catalog No. HY-11109, MedChemExpress) or lipopolysaccharides (LPS) (1 µg/mL, Catalog No. L4391, Sigma-Aldrich) for 48 h with or without liraglutide. As a TLR4 signaling inhibitor, TAK242 is used to inhibit the expression of the TLR4 signaling pathway, whereas LPS, as a TLR4 signaling pathway agonist, can specifically activate the TLR4 signaling pathway. For analysis, cell supernatants and lysates were collected and used for enzyme-linked immunosorbent assay (ELISA) and Western blotting, respectively.

#### In vivo study.

Wild-type (WT) C57BL/10 mice (males, 7–8 wk of age) and C57BL/10ScN mice (TLR4^−/−^ mice) (males, 7–8 wk of age) were purchased from the Model Animal Research Center of Nanjing University. After an overnight fast, diabetes was induced by intraperitoneally injecting multiple low doses of streptozotocin (STZ; Sigma; 50 mg/kg body wt daily for 5 days) dissolved in a citrate buffer (0.1 M, pH 4.5) (19, 20). Nondiabetic control mice received an equal volume of citrate buffer. Five days after STZ injection, mice with a fasting blood glucose (FBG) level (measured with a glucometer, Johnson) greater than 16.7 mmol/L for three consecutive days were identified as diabetic mice. Diabetic mice then received a normal diet for another 8 wk. The mice were divided into the following six groups: WT nondiabetic control (WT, *n* = 6), WT diabetes mellitus (DM) (WT + STZ, *n* = 6), WT DM treated with liraglutide (WT + STZ + LR, *n* = 6), TLR4^−/−^ nondiabetic control (TLR4^−/−^, *n* = 6), TLR4^−/−^ DM (TLR4^−/−^ + STZ, *n* = 6), and TLR4^−/−^ DM treated with liraglutide (TLR4^−/−^ + STZ + LR, *n* = 6). Mice in the DM plus liraglutide-treated group received subcutaneous injection with liraglutide at 200 μg/kg/12 h for 8 wk. However, mice in the DM group were given the same volume of physiological saline solution through the same route at the same frequency. All animal experimental protocols were approved by the Ethics Committee of Animal Research of Fujian Medical University.

### Transient Transfection with siRNAs and cDNA

The small interfering RNA (siRNA) duplexes corresponding to TLR4 (sense 5′-CCUUGGUACUGACAGGAAATT-3′; antisense 5′-UUUCCUGUCAGUACCAAGGTT-3′) and normal control siRNA (sense 5′-UUCUCCGAACGUGUCACGUTT-3′; antisense 5′-ACGUGACACGUUCGGAGAATT-3′), as well as the TLR4 overexpression plasmid pcDNA3.1-TLR4 (mRNA NCBI Reference Sequence: NM_019178.1) and empty pcDNA3.1 vector were purchased from GenePharma (Shanghai, PR China). Cells (5 × 10^5^ cells/well, in six-well plate) were plated to reach 60% to 80% confluence and transfected with siRNA (75 nM) and cDNA (3 μg) using Lipofectamine 2000 (6 μL, Invitrogen, Carlsbad, CA) according to the manufacturer’s instructions. At 48 h after transfection, Western blot was performed to determine transfection efficiency.

### Enzyme-Linked Immunosorbent Assay

The supernatant of RMCs after different treatments and kidney cortex of mouse were collected, the contents of IL-6, tumor necrosis factor-α (TNF-α), and monocyte chemotactic protein-1 (MCP-1) were measured by commercial ELISA kits (Bioss Biotechnology Co., Beijing, PR China) in accordance with the manufacturer’s instructions. Each group of an experiment was repeated four times. The absorbance value at 450 nm was determined using a microplate spectrophotometer.

### Western Blot

Cell and renal cortex protein extracts were prepared using the Total Protein Extraction Kit (TransGen Biotechnology Co., Beijing, PR China). Then the protein concentration of the cell extracts was measured using a bicinchoninic acid (BCA) protein assay kit (CW Biotechnology Co., Beijing, PR China). The cell lysates were electrophoresed on 8% SDS-PAGE and transferred to PVDF membranes. The membrane was blocked with 5% skim milk for 1 h at room temperature and probed by primary antibodies at 4°C overnight. The antibodies and the optimal dilutions used in this study were as follows: FN (Catalog No. ab2413, 1:1,000, Abcam), collagen type IV (Col IV; Catalog No. ab135802, 1:100, Abcam), α-SMA (Catalog No. 19245, 1:1,000, Cell Signaling Technology), α-tubulin (Catalog No. sc-5286, 1:500, Santa Cruz Biotechnology), TLR4 (Catalog No. sc-293072, 1:500, Santa Cruz Biotechnology), myeloid differentiation marker 88 (MyD88; Catalog No. ab2064, 1:1,000, Abcam), NF-κB p65 (Catalog No. GB11142-1, 1:1,000, Servicebio), and NOD-like receptor family pyrin domain containing 3 (NLRP3; Catalog No. ab214185, 1:300, Abcam). Then the proteins were probed by incubating with horseradish peroxidase (HRP)-conjugated secondary antibody for 1 h at 37°C. The integrated density values of the target protein bands were measured by AlphaImage 2000 software (Alpha Innotech, San Leandro, CA) for semiquantitative analysis. Data are presented as fold-induction normalized to α-tubulin.

### Sample Collections and Urinary Albumin Assessment

The 24-h urine output of all mice was measured after 8 wk of treatment. Urine samples were collected using metabolic cages. All mice were euthanized by intraperitoneal injection of pentobarbital sodium (100 mg/kg) after the experiments. Kidneys were dissected and weighed. Urine was collected over 16 h on the day before euthanasia. Kidney tissues were harvested at time of euthanasia. The left kidney was fixed with 10% neutral-buffered formalin for paraffin embedding, and the right kidney was quickly frozen in liquid nitrogen to harvest for Western blotting and ELISA. Urinary albumin and creatinine excretion was determined using an Albumin-to-Creatinine Ratio Assay Kit (BioVision).

### Renal Histology

The left kidney was sliced longitudinally into 3- to 5-μm sections and stained with hematoxylin and eosin (H&E), periodic acid-Schiff (PAS), and Masson trichrome (Masson) to assess renal pathology. The glomerular area and mesangial matrix index were calculated using Image J software as previously described (21). Masson trichrome-positive staining area percentage was calculated using Image-Pro Plus 6.0 analysis software. Three sections were randomly selected from the left kidney of one mouse in each group, and at least six glomeruli per section were used for histomorphometric analysis.

### Immunohistochemistry

Left kidney sections were incubated with primary antibodies against FN, Col IV, α-SMA, α-tubulin, TLR4, MyD88, and NF-κB p65 for 1 h at room temperature, followed by incubation with horseradish peroxidase-conjugated antirabbit/mouse secondary antibody (Zhongshan Jinqiao Biotechnology Co., Beijing, PR China) for 30 min at room temperature. For the negative control, sections were incubated with PBS instead of primary antibody. The sections were counterstained with hematoxylin after being stained with diaminobenzidine (DAB) to produce a brown-colored precipitate. The sections were observed by light microscopy. The average integrated optical density (magnification: ×400) of the individual protein staining was analyzed using Image-Pro Plus image-analysis software. Six glomeruli per section from three sections per kidney per mouse in each group were used in this experiment.

### Statistical Analysis

All values were expressed as means ± standard deviation (SD) calculated from at least five repeated experiments. Differences among groups were analyzed by one-way ANOVA followed by a Student–Newman–Keuls test for multiple comparisons. A value of *P* < 0.05 was considered statistically significant, and values of <0.01 were considered highly significant. Statistical analysis was performed using IBM SPSS Statistics 22 software.

## RESULTS

### Liraglutide Reduced the Excessive Secretion of ECM Protein and Inflammatory Factors in RMCs Induced by HG

**Figure 1.**
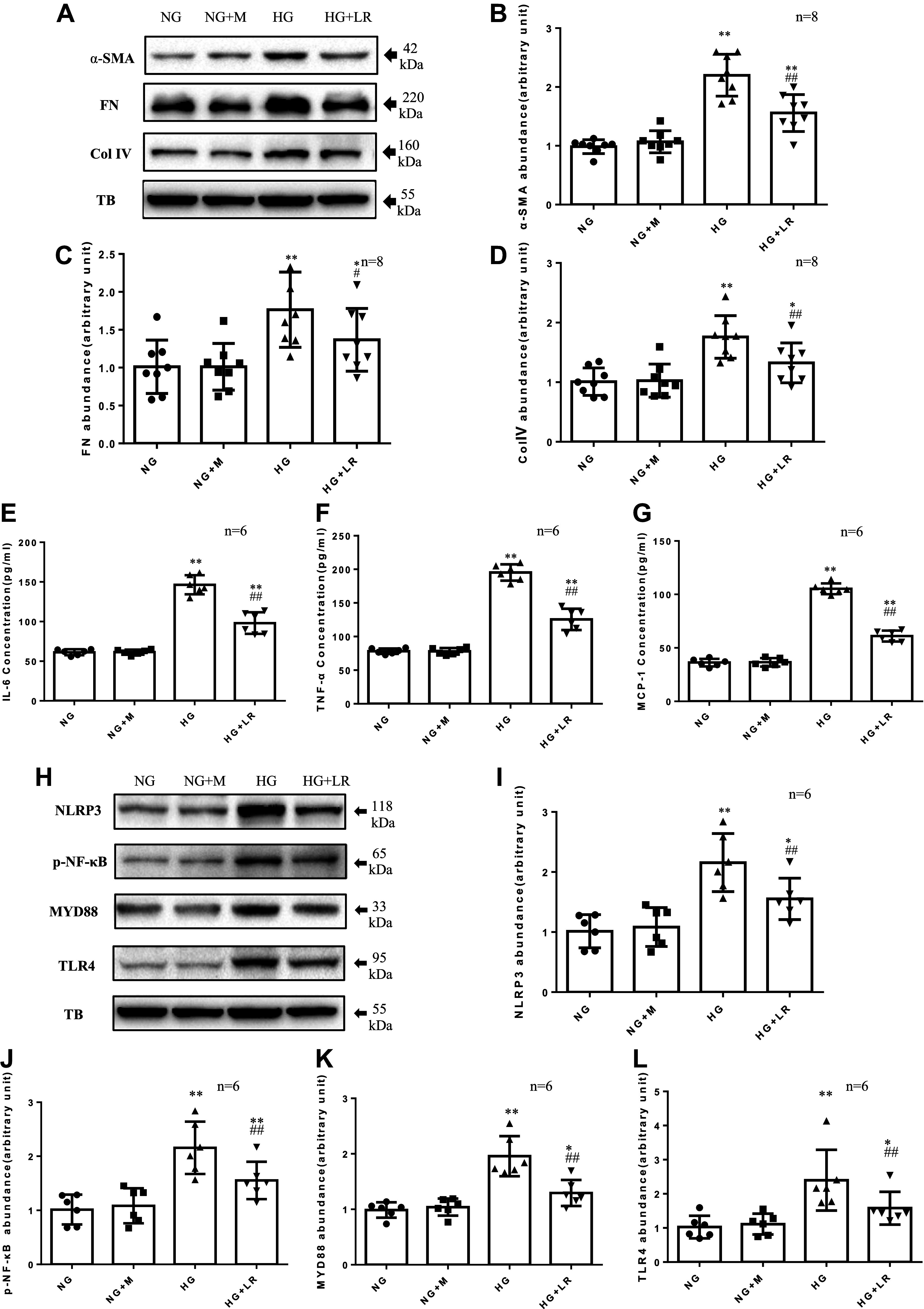
Liraglutide reduced high glucose (HG)-induced increases of Toll-like receptor (TLR)4/myeloid differentiation marker 88 (MyD88)/nuclear factor κB (NF-κB) pathway, extracellular matrix (ECM), and inflammatory factors in rat glomerular mesangial cell lines (RMCs). RMCs were incubated in media containing 5.6 mM d-glucose (normal glucose, NG) or 5.6 mM d-glucose + 20 mM mannitol (NG + M) or 25 mM d-glucose (HG) with or without 100 nM liraglutide (LR). The protein levels of α-smooth muscle actin (α-SMA), fibronectin (FN), collagen type IV (Col IV), NOD-like receptor family pyrin domain containing 3 (NLRP3), NF-κB, MyD88, and TLR4 were measured by Western blot (*A*–*D*, *H*–*L*). The protein levels of interleukin-6 (IL-6), tumor necrosis factor-α (TNF-α), and monocyte chemotactic protein-1 (MCP-1) were measured by ELISA (*E*–*G*). TB, α-tubulin. *P < 0.05, **P < 0.01 compared with both NG and NG + M groups. #*P* < 0.05, ##*P* < 0.01 vs. HG group. *n*, no. of independent experiments.

Our previous study found that liraglutide and exendin-4 reduced HG-induced ECM protein oversecretion in human MCs by enhancing the expression of GLP-1 receptors and presented a certain concentration dependence (10). In the present study, as shown in Fig. 1, *A*–*G*, HG stimulated RMCs to excessively secrete matrix proteins (FN and Col IV), α-SMA, and inflammatory cytokines (IL-6, TNF-α, and MCP-1), whereas 100 nM liraglutide significantly reduced the secretion of ECM proteins and inflammatory cytokines in RMCs. These data, consistent with published studies (10, 21, 22), indicate that GLP-1RA reduces abundance of ECM and α-SMA proteins and correspondingly, decreases chronic inflammatory factors in MCs under diabetic conditions.

### Liraglutide Reduced HG-Induced Activation of TLR4/MYD88/NF-κB/NLRP3 Signaling Pathway in RMCs

At present, TLR4/MyD88/NF-κB pathway is considered to be the main factor that promotes chronic inflammation and fibrosis in kidney (14, 15). To investigate whether the anti-inflammatory and antifibrotic effects of GLP-1RA on MCs are associated with the TLR4/MyD88/NF-κB pathway, several key proteins in the TLR4/MyD88/NF-κB pathway were detected by Western blotting. As shown in Fig. 1, *H*–*L*, HG significantly increased the expression of TLR4, MyD88, NF-κB, and NLRP3. However, treatment with 100 nM liraglutide significantly reduced the overexpression of TLR4/MyD88/NF-κB/NLRP3 signaling proteins induced by HG. These results suggest that GLP-1RA is involved in regulating the TLR4 signaling pathway in MCs.

### Effect of Liraglutide with and without Inhibition or Activation of TLR4 on HG-Induced ECM Protein Abundance and Inflammatory Factors Production in RMCs

**Figure 2.**
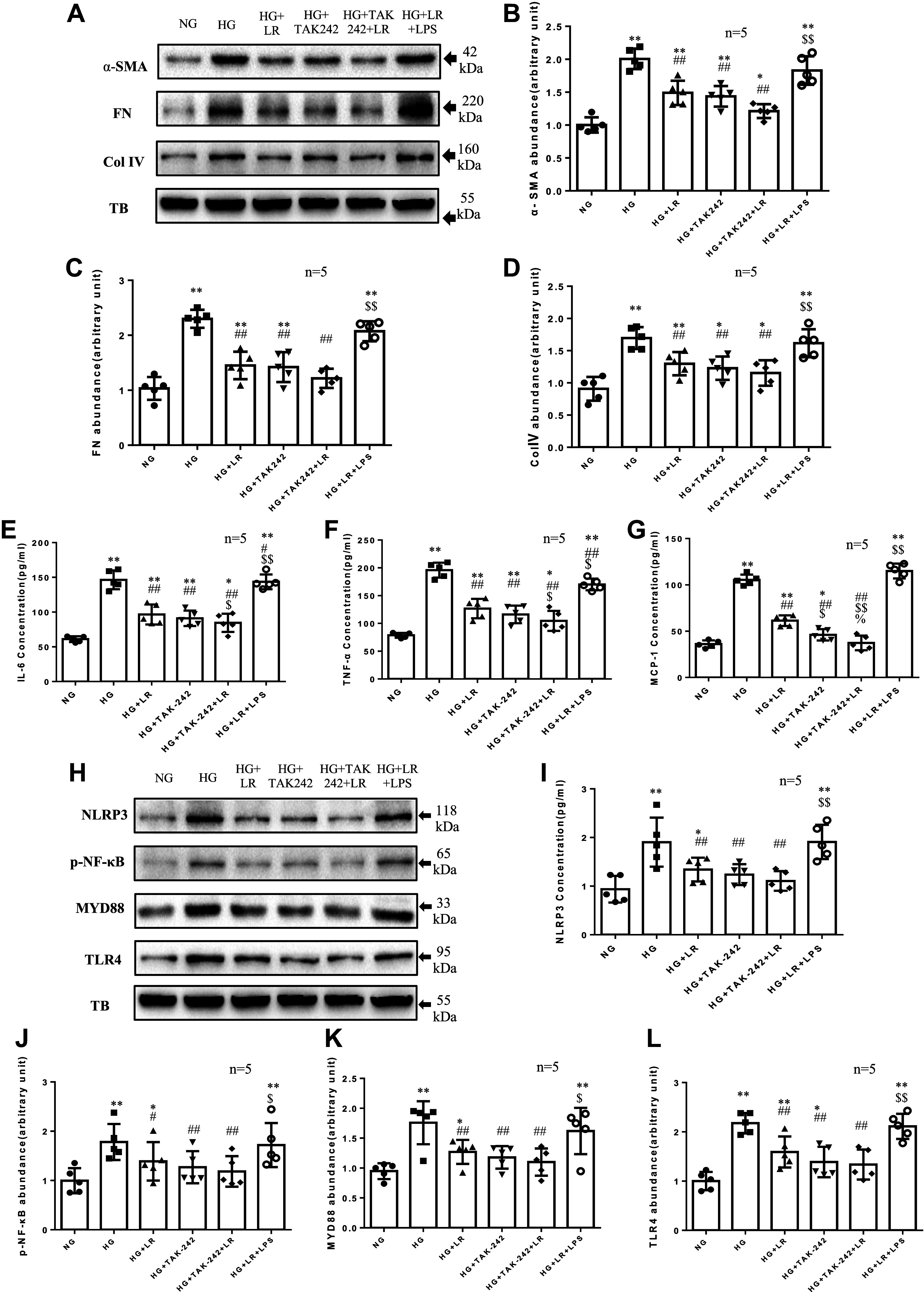
Toll-like receptor (TLR)4 agonist and inhibitor influence the effects of liraglutide (LR) on high glucose (HG)-induced extracellular matrix (ECM) protein abundance and inflammatory factors production in rat glomerular mesangial cell lines (RMCs). RMCs were incubated in media containing 5.5 mM glucose (normal glucose, NG) or HG in the absence or presence of 100 nM liraglutide (LR) or TAK242 (1 µM) or lipopolysaccharides (LPS, 1 µg/mL). The protein levels of α-smooth muscle actin (α-SMA), fibronectin (FN), collagen type IV (Col IV), NOD-like receptor family pyrin domain containing 3 (NLRP3), nuclear factor κB (NF-κB), myeloid differentiation marker 88 (MyD88), and TLR4 were measured by Western blot (*A*–*D*, *H*–*L*). The protein levels of interleukin-6 (IL-6), tumor necrosis factor-α (TNF-α), and monocyte chemotactic protein-1 (MCP-1) were measured by ELISA (*E*–*G*). TB, α-tubulin. *P < 0.05, **P < 0.01 compared with NG groups. #*P* < 0.05, ##*P* < 0.01 vs. HG group. $*P* < 0.05, $$*P* < 0.01 vs. HG + LR group. %*P* < 0.05. *n*, no. of independent experiments.

To determine whether the improvement effect of liraglutide on the HG-induced production of inflammatory factors and ECM proteins is related to TLR4/MyD88/NF-κB signaling pathway in RMCs, we conducted two lines of experiments. First, we used TLR4 inhibitor (TAK242) or agonist (LPS) to inhibit or activate the TLR4 signaling pathway and examined the influence on the liraglutide response. As presented in Fig. 2, TAK242 decreased the expression of TLR4/MYD88/NF-κB/NLRP3 signaling protein induced by HG and decreased HG-induced ECM proteins and inflammatory factors. In the presence of TAK242, liraglutide could not further improve TLR4/MYD88/NF-κB signaling, inflammatory factors, and ECM proteins. Interestingly, in the presence of the TLR4 agonist LPS, the improvement effect of liraglutide on the TLR4/MYD88/NF-κB signaling pathway, ECM, and inflammatory factors was completely abolished.

**Figure 3.**
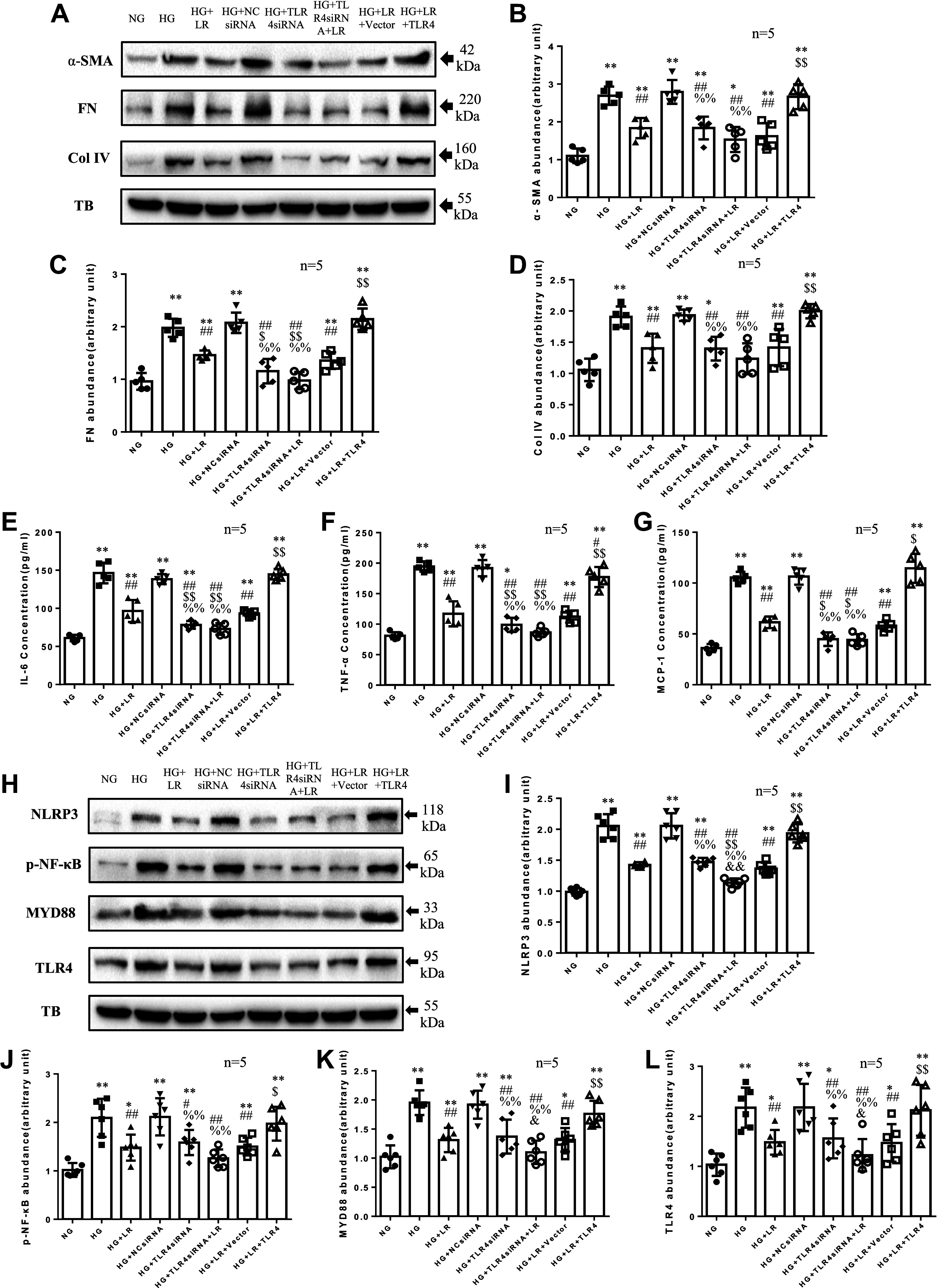
Effects of liraglutide (LR) with or without Toll-like receptor (TLR)4 silence or overexpression on high glucose (HG)-induced extracellular matrix (ECM) protein abundance and inflammatory factors production in rat glomerular mesangial cell lines (RMCs). RMCs were incubated in media containing 5.5 mM glucose (normal glucose, NG) or HG in the absence or presence of 100 nM liraglutide (LR), without transfection or transfected with normal control siRNA (NCsiRNA) or TLR4 siRNA or empty pcDNA3.1 vector (Vector) or TLR4 expression plasmid pcDNA3.1-TLR4 (TLR4). The protein levels of α-smooth muscle actin (α-SMA), fibronectin (FN), collagen type IV (Col IV), NOD-like receptor family pyrin domain containing 3 (NLRP3), nuclear factor κB (NF-κB), myeloid differentiation marker 88 (MyD88), and TLR4 were measured by Western blot (*A*–*D*, *H*–*L*). The protein levels of interleukin-6 (IL-6), tumor necrosis factor-α (TNF-α), and monocyte chemotactic protein-1 (MCP-1) were measured by ELISA (*E*–*G*). TB, α-tubulin. *P < 0.05, **P < 0.01 compared with NG groups. #*P* < 0.05, ##*P*< 0.01 vs. HG group. $*P* < 0.05, $$*P*< 0.01 vs. HG + LR and HG + LR + Vector group. %%*P* < 0.01 vs. HG + NCsiRNA group. &*P* < 0.05, &&*P* < 0.01 vs. HG + TLR4siRNA group. *n*, no. of independent experiments.

Second, to further verify that these effects of liraglutide are closely related to the TLR4/MYD88/NF-κB signaling pathway, we manipulated TLR4 function using biological approaches. Overexpression of TLR4 was achieved by transfection with TLR4 expression plasmid. TLR4 knockdown was done using siRNA approach. As shown in Fig. 3, TLR4 knockdown decreased the expression of TLR4/MYD88/NF-κB/NLRP3 signaling, and decreased HG-induced ECM proteins and inflammatory factors, similar to those observed in liraglutide-treated RMCs. Furthermore, in the presence of TLR4 siRNA, liraglutide significantly blunted HG-induced expression of TLR4/MYD88/NF-κB/NLRP3 signaling protein and Col4, SMA, similar to the effect of liraglutide without inhibition of TLR4. Regarding FN protein and inflammatory factors, the effect of liraglutide with TLR4 knockdown was greater than without inhibition of TLR4. However, TLR4 overexpression completely abolished the inhibitory effects of liraglutide on the HG-induced response. These results indicated that GLP-1RA liraglutide reduces HG-induced overproduction of ECM and inflammatory factors through downregulation of the TLR4/MYD88/NF-κB/NLRP3 pathway.

### Effects of Liraglutide Treatment on Urinary Protein Excretion, Glomerular Histopathological Changes, and Fibrosis in TLR4^−/−^ DM Mice

**Figure 4.**
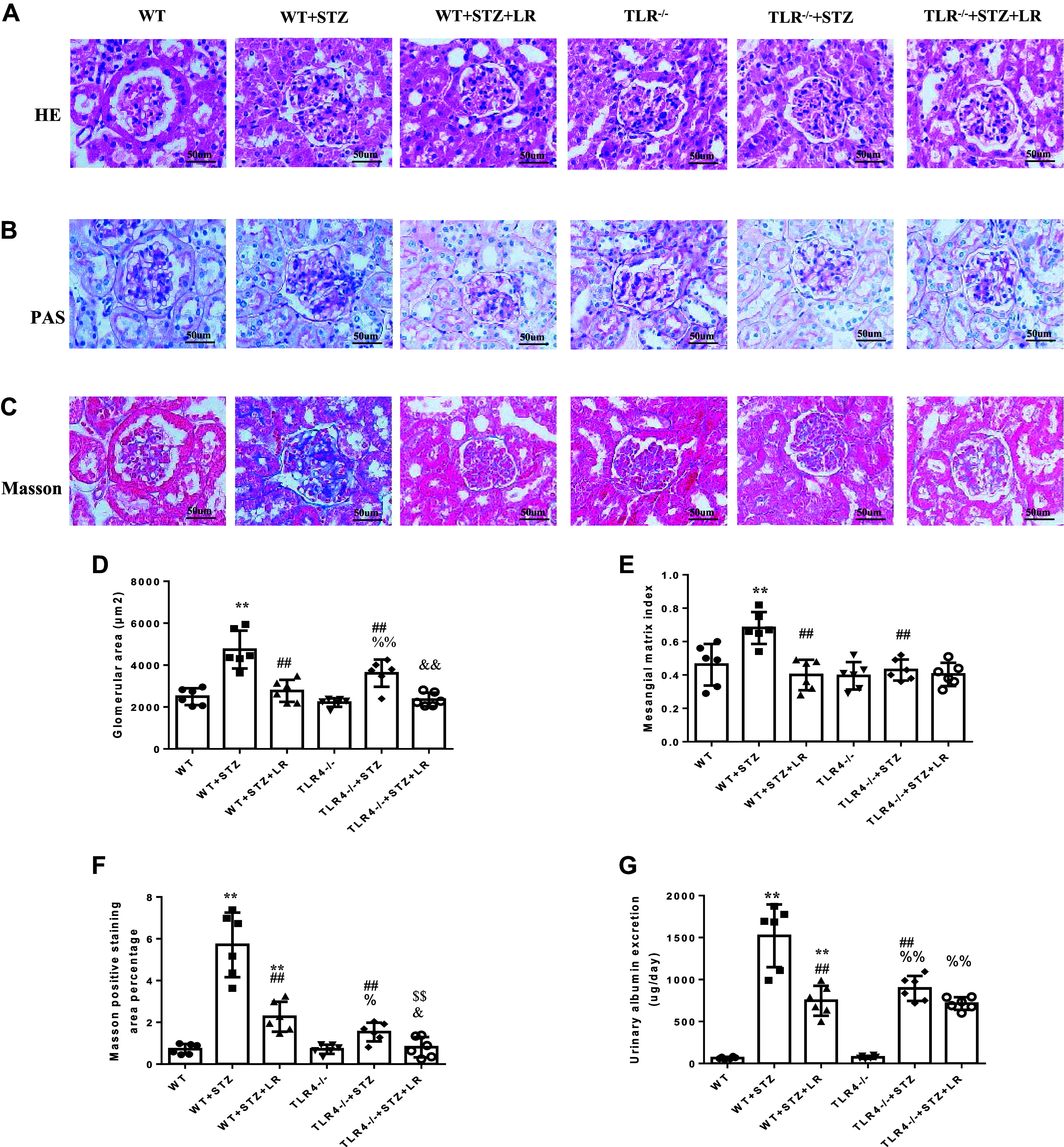
Effects of liraglutide treatment on urinary protein excretion, glomerular histopathological changes, and fibrosis in Toll-like receptor (TLR)4^−/−^ diabetes mellitus (DM) mice. *A*–*C*: representative photomicrographs of hematoxylin and eosin (H&E) staining (*A*), periodic acid-Schiff (PAS) staining (*B*), and Masson trichrome staining (*C*) of renal cortex sections from six groups of mice. *D*–*F*: quantitative analysis of H&E staining (*D*), PAS staining (*E*), and Masson trichrome staining (*F*). *G*: urinary albumin creatinine. LR, liraglutide; TLR4^−/−^, TLR4^−/−^ mice; WT, wild-type mice; WT + STZ, streptozotocin-treated WT mice. **P < 0.01 compared with WT groups. ##*P*< 0.01 vs. WT + STZ group. $$*P* < 0.01 vs. WT + STZ + LR group. %%*P* < 0.01 vs. TLR4^−/−^ group. &*P* < 0.05, &&*P* < 0.01 vs. TLR4^−/−^ + STZ group. Magnification: ×400.

As presented in Fig. 4, histopathological examination of renal tissue showed glomerular hypertrophy, mesangial expansion on PAS staining in STZ-treated WT mice. In addition, Masson staining showed large amount of collagen deposition in interstitial space of glomeruli and tubules in STZ-induced diabetic nephropathy (DN) mice. The excretion of urinary protein was also significantly increased in mice with DN. As expected, in liraglutide-treated DN mice, these pathological changes improved significantly, and the protein excretion in urine was also significantly reduced. Next, we used TLR4^−/−^ mice to observe the role of TLR4 in liraglutide’s kidney protection. Compared with WT mice, TLR4^−/−^ mice had no significant differences in urine protein excretion, glomerular shape, and fibrosis. In TLR4^−/−^ DM mice, the urine protein excretion, glomerular area, mesangial matrix index, and Masson positive staining area percentage were much less than STZ-treated WT mice. In addition, compared with TLR4^−/−^ mice, TLR4^−/−^ DM mice showed significantly increased glomerular hypertrophy, Masson-positive staining area percentage, and urinary protein excretion. Moreover, liraglutide treatment modestly improved mesangial expansion and glomerular fibrosis in STZ-induced TLR4^−/−^ DM mice. In addition, compared with DM mice treated with liraglutide, the degree of glomerular fibrosis in the TLR4^−/−^ DM mice treated with liraglutide decreased to a certain extent, which was statistically significant.

### Effects of Liraglutide Treatment on ECM Accumulation, Inflammatory Factors, and TLR4/MYD88/NF-κB Signaling Protein in Glomeruli of TLR4^−/−^ DM Mice

**Figure 5.**
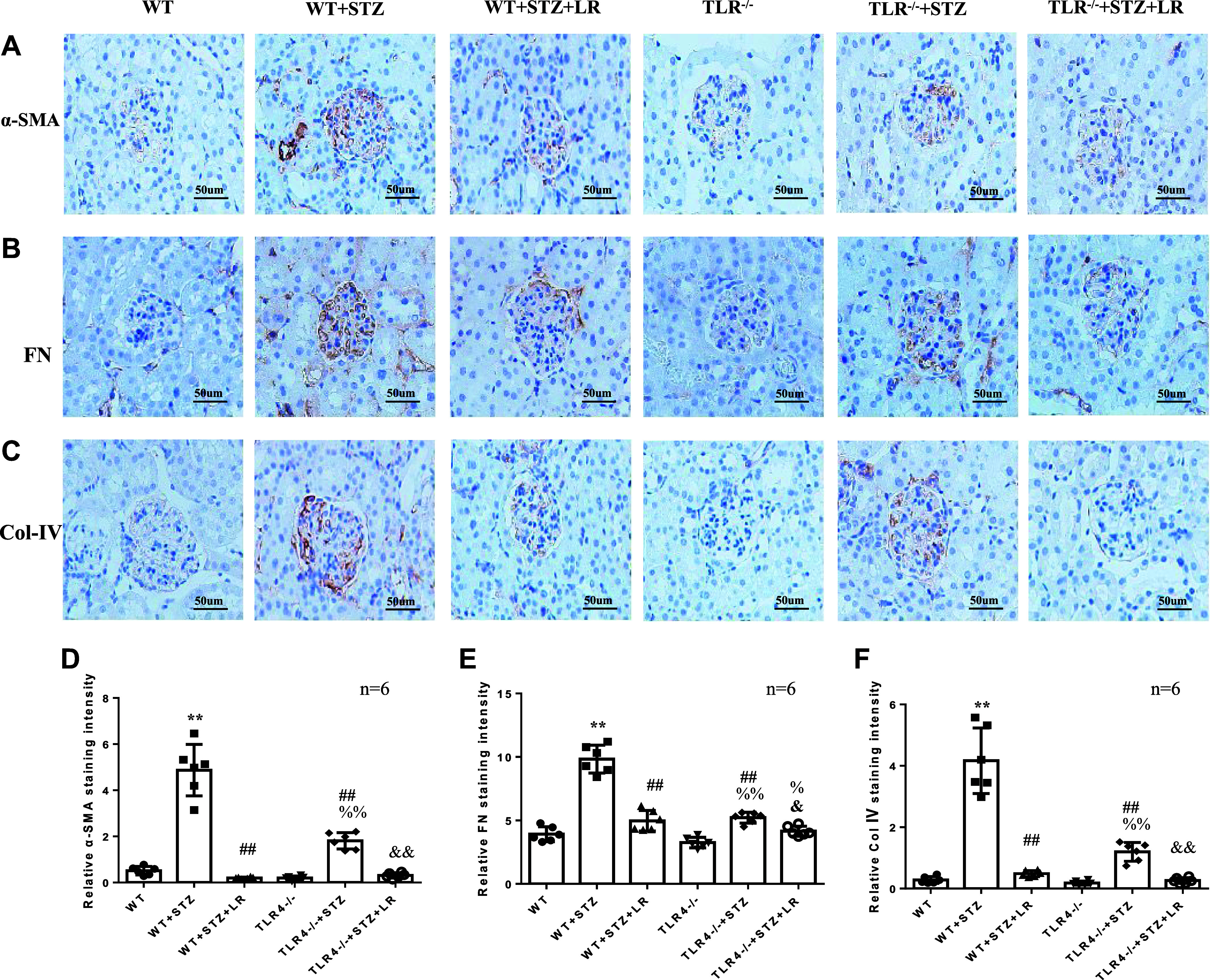
Effects of liraglutide (LR) treatment on glomerular extracellular matrix (ECM) in Toll-like receptor (TLR)4^−/−^ diabetes mellitus (DM) mice by immunohistochemistry. *A*–*C*: representative immunohistochemical staining images of α-smooth muscle actin (α-SMA, *A*), fibronectin (FN, *B*), and collagen type IV (Col IV, *C*) in glomeruli from six groups of mice. *D*–*F*: semiquantitative analysis of staining intensity of α-SMA (*D*), FN (*E*), and Col IV (*F*). WT, wild-type mice; WT + STZ, streptozotocin-treated WT mice. **P < 0.01 compared with WT groups. ##*P*< 0.01 vs. WT+STZ group. %*P* < 0.05, %%*P* < 0.01 vs. TLR4^−/−^ group. &*P* < 0.05, &&*P* < 0.01 vs. TLR4^−/−^ + STZ group. Magnification: ×400. *n*, no. of mice in each group.

**Figure 6.**
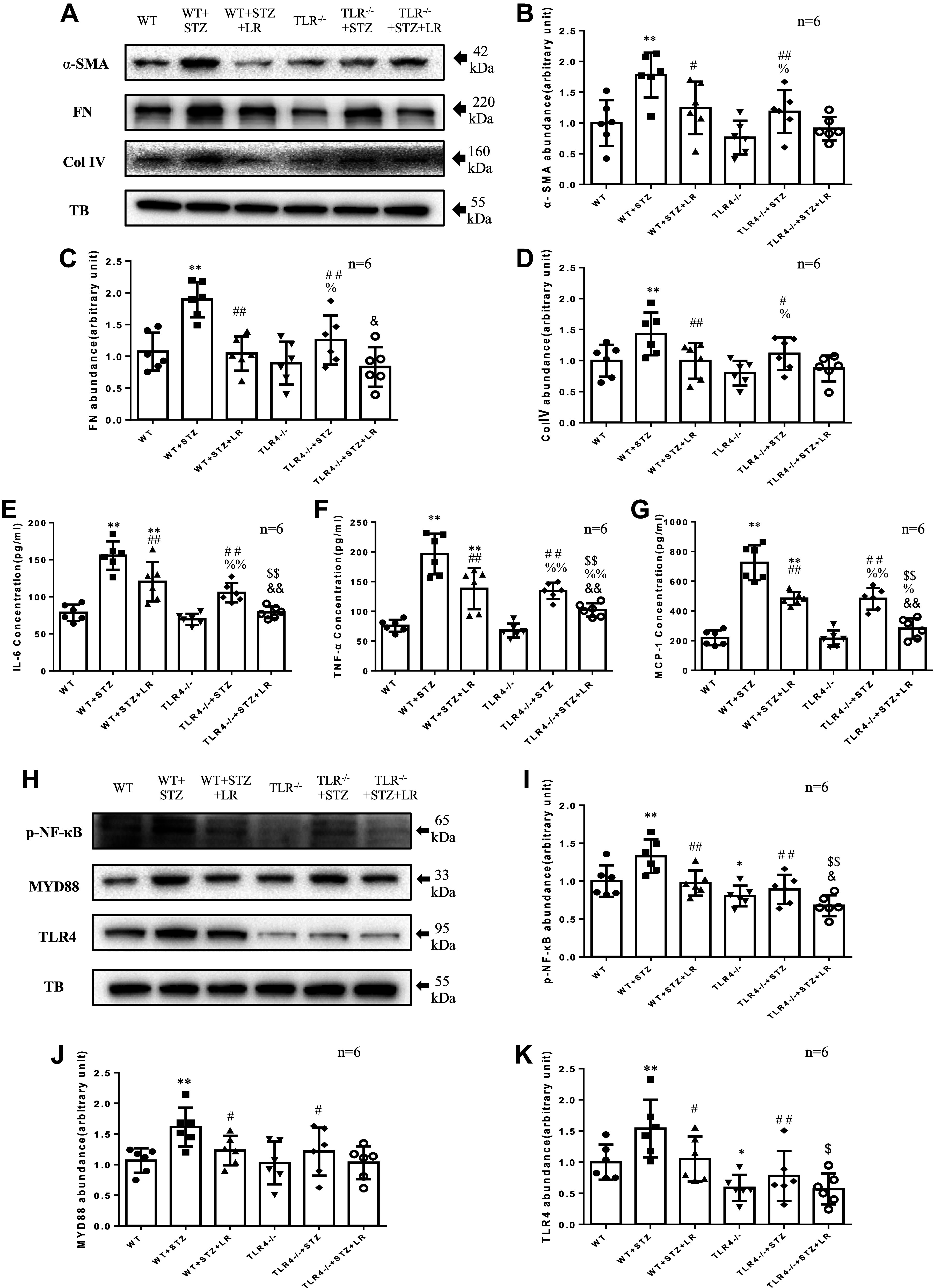
Effects of liraglutide (LR) treatment on glomerular Toll-like receptor (TLR)4/myeloid differentiation marker 88 (MyD88)/nuclear factor κB (NF-κB) signaling pathway, extracellular matrix (ECM) accumulation, and inflammatory factors in TLR4^−/−^ diabetes mellitus (DM) mice. The protein levels of α-smooth muscle actin (α-SMA), fibronectin (FN), collagen type IV (Col IV), NF-κB, MYD88, and TLR4 were measured by Western blot (*A*–*D*, *H*–*K*). The protein levels of interleukin-6 (IL-6), tumor necrosis factor-α (TNF-α), and monocyte chemotactic protein-1 (MCP-1) were measured by ELISA (*E*–*G*). TB, α-tubulin. *P < 0.05, **P < 0.01 compared with wild-type (WT) groups. #*P* < 0.05, ##*P* < 0.01 vs. WT + streptozotocin (STZ) group. $*P* < 0.05, $$*P* < 0.01 vs. WT + STZ + LR group. %*P* < 0.05, %%*P* < 0.01 vs. TLR4^−/−^ group. &*P* < 0.05, &&*P* < 0.01 vs. TLR4^−/−^ + STZ group. Magnification: ×400. *n*, no. of mice in each group.

The levels of ECM proteins were checked by immunohistochemical and Western blotting analysis, and several inflammatory factors by ELISA. As shown in Fig. 5 and Fig. 6, *A*–*G*, DN significantly increased glomerular FN, Col IV, α-SMA, and inflammatory factors (IL-6, TNF-α, and MCP-1). However, the fibrosis and inflammation in DN mice were significantly reduced after liraglutide treatment. The improvement of renal fibrosis and inflammation by deletion of TLR4 was further indicated by significantly less increase in ECM protein and inflammatory factors in TLR4^−/−^ diabetic mice compared with WT diabetic mice. Liraglutide treatment decreased IL-6, TNF-α, and MCP-1 in TLR4^−/−^ DM mice. Immunohistochemical analysis showed FN, Col IV, and α-SMA expression decreased significantly, while Western blotting analysis showed a significant decrease in FN in TLR4^−/−^ DM mice treated with liraglutide.

Furthermore, the expression levels of glomerular TLR4, MYD88, and NF-κB were significantly increased in DN mice compared with WT nondiabetic mice (Fig. 6, *H*–*K*), indicating that the TLR4/MYD88/NF-κB signaling pathway was activated in DN mice. The TLR4/MYD88/NF-κB signaling pathway was significantly decreased after treatment with liraglutide. Compared with TLR4^−/−^ mice, there were no statistical differences in the expression levels of TLR4, MYD88, and NF-κB in TLR4^−/−^ diabetic mice. After liraglutide intervention, NF-κB decreased in TLR4^−/−^ DM mice. In addition, NF-κB was significantly decreased in liraglutide-treated TLR4^−/−^ DM mice compared with liraglutide-treated DM mice.

In summary, the data from animal experiments were consistent with the results from cultured cells, further indicating that liraglutide reduces glomerular inflammation and overproduction of ECM by downregulating TLR4/MYD88/NF-κB signaling under diabetic conditions.

## DISCUSSION

In the present study, liraglutide treatment decreased the expression of TLR4/MYD88/NF-κB/NLRP3 signaling protein, thereby reducing the inflammatory response and overproduction of ECM protein in MCs induced by HG. In vivo, liraglutide downregulated TLR4/MYD88/NF-κB signaling, and improved the urinary protein excretion rate, glomerular pathological damage, inflammation, and fibrosis in DN mice. TLR4^−/−^ diabetic mice showed significant amelioration in urine protein excretion rate, glomerular pathological damage, inflammation, and fibrosis.

Recent studies have shown that GLP-1RA still has a renal protective effect without lowering blood sugar levels. For example, liraglutide and exetide-4 reduced proteinuria and alleviated renal oxidative stress and inflammation in STZ-induced type 1 diabetes rats without reducing blood glucose levels (23, 24). Similar findings have been reported in KK/Ta Akita mice and *db/db* mice treated with GLP-1RA (25–27). Our previous research has shown that liraglutide significantly improves kidney injury and fibrosis in a glucose-independent manner in rats with DN (21). Therefore, to avoid the effect of lowering blood glucose on the experiment, we chose to use STZ-induced type 1 diabetes mice to explore the role of GLP-1RA in DKD.

Activation of the TLR4/MYD88/NF-κB signaling pathway is one of the important mechanisms leading to impaired renal function. Kaur et al. (28) reported that hyperglycemia induced the expression and activity of TLR4 in RMCs and stimulated the activation of MyD88, interferon regulatory factor 3, and NF-κB, thereby increasing the secretion of inflammatory factors. In STZ-induced DN mice, the glomeruli showed higher expression of TLR4 and inflammatory cytokines (IL-6 and TNF-α) (29). In the present study, HG/DM increased the TLR4/MYD88/NF-κB signaling proteins in MCs/glomerulus, and the expression of proinflammatory cytokines (IL-6, TNF-α, and MCP-1), α-SMA, and ECM proteins (FN and Col IV) both in vivo and in vitro. Thus, TLR4 signaling, inflammatory factors, and ECM proteins in MCs are significantly activated in the HG state. Therefore, the excessive activation of the TLR4 pathway plays a crucial role in maintaining the inflammatory factors and ECM proteins overproduced by MCs in DKD. In nondiabetic-induced renal damage animal models, whether it is unilateral ureteral obstruction (UUO) model mice or cyclosporin-induced renal impairment mice, TLR4^−/−^ mice showed mild inflammatory response as observed in the present study (30, 31). In the STZ-induced type 1 DN model, TLR4^−/−^ DM mice showed a significant reduction of Myd88 and NF-κB expression with decreased levels of inflammatory and fibrosis factors, thus improving the renal injury (32). Similar results were found in our experiments. In vitro, inhibition of TLR4 by siRNA or TAK242 (TLR4 inhibitor) significantly reduced the expression of MYD88, NF-κB, NLRP3, inflammatory factors, and fibrosis-related factors. In vivo, STZ-induced TLR4^−/−^ mice showed lessened renal injury compared with STZ-treated WT mice. These included decreased albuminuria, mesangial matric expansion, and glomerular fibrosis, decreased abundance of matrix proteins, and inflammatory factors. Therefore, downregulation of the TLR4/MYD88/NF-κB pathway can delay the fibrosis and inflammatory response of DKD, which also provides a new idea for the treatment of DKD.

In STZ-induced diabetes, multiple inflammatory factors are involved. The GLP-1RA exendin-4 treatment effectively lessened the overexpression of a variety of inflammatory biomarkers, and improved the histological characteristics of DKD (33), and this phenomenon was still valid in type 1 diabetic mouse model (24). Besides MCs, GLP-1RA also have protective effects on other renal cells. Ye et al. (34) found that liraglutide alleviated the morphological and structural damage of podocytes in obesity-related nephropathy mice, and reduced TNF-α expression and activation of NF-κB. In renal tubular epithelial cells, exendin-4 improves HG-induced FN and collagen I, thereby improving renal fibrosis (35). However, in DKD, whether the effect of GLP-1RA on improving fibrosis and inflammation is related to the downregulation of the TLR4 signaling pathway is still unknown.

The TLR4 signaling is involved in the protective effects of several GLP-1RA on organs/tissues outside the kidney (16, 36, 37). Xu et al. (16) reported that a combination of liraglutide and human umbilical cord mesenchymal stem cells could significantly decrease the expression of inflammatory factors and improve oxidative stress by downregulating the TLR4/NF-κB signaling pathway in rats with T2DM/nonalcoholic fatty liver disease. In patients with obesity with T2DM, liraglutide exerted anti-inflammatory effect by lessening TLR4, NF-κB and downregulating the expression of proinflammatory factors including TNF-α (37). In a lethal renal ischemia-reperfusion injury model, liraglutide improved the survival rate, reduced renal pathological injury, and decreased the mRNA levels of TLR4, TNF-α, IL-1β, IL-6, and MCP-1 (38). In the renal injury model induced by angiotensin II, after treatment with liraglutide or dipeptidyl peptidase-4 inhibitor linagliptin, rat glomeruli TLR4, TGFβ1, phosphorylated Smad2/3, and Smad4 expressions were significantly reduced, whereas the macrophages and α-SMA-expressing myofibroblasts were significantly decreased, thereby improving the fibrosis of the kidney (9). However, the effect of GLP-1RA on the expression of TLR4 in MCs under HG conditions and whether the effect of GLP-1RA on reducing inflammation and fibrosis in DKD is related to the TLR4 pathway are still unclear. In the present study, liraglutide treatment downregulated the TLR4/MYD88/NF-κB/NLRP3 signaling pathway, proinflammatory cytokines, and fibrosis-related markers in MCs under a HG environment. Importantly, blocking TLR4 also lessened HG-induced inflammatory response and excessive ECM protein secretion, and the downregulatory effect of liraglutide on inflammatory response and ECM accumulation was significantly abolished after upregulating TLR4 expression. In vivo, the treatment with liraglutide blocked the upregulation of glomerular TLR4/MYD88/NF-κB signaling pathways, decreased proinflammatory cytokines and fibrosis-related indicators in the DN mouse model, and achieved regression of glomerular fibrosis. The combination of these results indicated that the TLR4 signaling pathway mediated, at least partially, the renal protective mechanism of liraglutide in DKD.

Our study found that liraglutide combined with TLR4 inhibitor/siRNA did not yield a stronger synergistic effect under HG conditions, including not further reducing the fibrosis and inflammatory response of MCs. Moreover, liraglutide still appropriately alleviated glomerular hypertrophy, renal fibrosis, and inflammatory response in TLR4^−/−^ DM mice. These results imply that other pathways are involved in the renoprotective effects of GLP-1RA. For instance, our previous studies have observed that GLP-1RA upregulated the expression of GLP-1 receptor and alleviated HG-stimulated ECM production through store-operated calcium entry (SOCE) and Wnt/β-catenin signaling in DN (10, 21). Other researchers also found that exendin-4 significantly attenuated the production of inflammatory cytokines (IL-6 and TNF-α) induced by advanced glycation end products in RMCs, which play a major role in the progression of DKD (22). Therefore, the renal protection of GLP-RA may involve multiple signaling pathways.

### Perspectives and Significance

**Figure 7.**
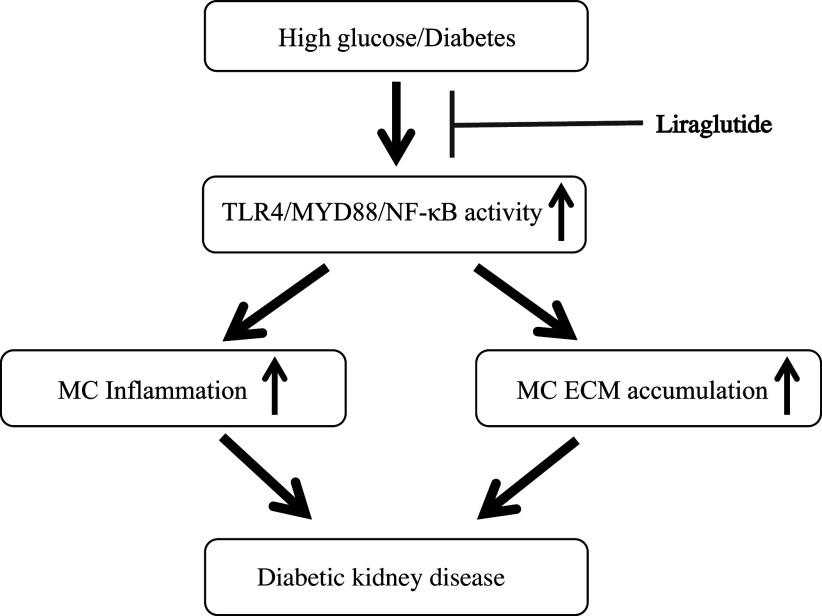
Illustration of liraglutide on inflammation and extracellular matrix (ECM) through the Toll-like receptor (TLR)4 signaling pathway. Schematic illustration of the mechanistic pathway mediating the liraglutide effect on mesangial cells (MCs) in diabetic kidney disease. High glucose or diabetes activates TLR4/myeloid differentiation marker 88 (MyD88)/nuclear factor κB (NF-κB) signaling pathway in MCs, resulting in extracellular matrix (ECM) protein production in diabetic kidney disease. Liraglutide downregulates TLR4/MyD88/NF-κB signaling, thus blunts high glucose-induced ECM protein accumulation and ameliorates diabetic kidney disease.

In summary, we demonstrated that GLP-1RA liraglutide alleviated glomerular inflammation and fibrosis in DKD at least partially by the TLR4 signaling pathway. This novel renal protection pathway is shown in Fig. 7. Because of the increasing number of patients with DKD around the world, there is an urgent need to find a better treatment. Our findings in this study may provide a new theoretical basis for the treatment strategies of DKD.

## DATA AVAILABILITY

Data will be made available upon reasonable request.

## GRANTS

Sponsorship for this study and Rapid Service Fee were funded by Joint Funds for the Innovation of Science and Technology, Fujian Province under Grant No. 2021Y9106 and Fujian Provincial Health Technology Project under Grant Nos. 2020CXA035 and 2021GGA033.

## DISCLOSURES

No conflicts of interest, financial or otherwise, are declared by the authors.

## AUTHOR CONTRIBUTIONS

L.H. conceived and designed research; L.H., T.L., and M.S. performed experiments; T.L. analyzed data; T.L. interpreted results of experiments; M.S. prepared figures; L.H. drafted manuscript; P.W. edited and revised manuscript; P.W. approved final version of manuscript.
